# ‘It’s not just immoral!’: The role of moral disengagement and incivility in dehumanising the transgressor of immoral behaviour

**DOI:** 10.1371/journal.pone.0322212

**Published:** 2025-05-07

**Authors:** Sofía Moreno-Gata, Ramón Rodríguez-Torres, Armando Rodríguez-Pérez, Verónica Betancor

**Affiliations:** Department of Cognitive, Social and Organizational Psychology, School of Psychology and Speech Therapy, University of La Laguna, San Cristóbal de La Laguna, Santa Cruz de Tenerife, Spain; International University - Vietnam National University Ho Chi Minh City, VIET NAM

## Abstract

People who engage in immoral behaviour are often dehumanised. However, they also tend to justify their actions to convince themselves or others that their misconduct is morally acceptable. In this paper, we examine whether moral disengagement mechanisms influence the extent to which a transgressor is perceived as fully human. Further, we assess whether this perception varies based on specific characteristics of the immoral behaviour, such as incivility. To answer these research questions, we conducted two studies with online participants from Spain. In Study 1, participants (*N* = 302) evaluated a set of 63 behaviours. For every behaviour, they assessed the extent to which it violated each moral foundation from moral foundations theory, its level of incivility, and its potential to elicit dehumanisation. A correlation analysis showed that only the care and purity foundations, along with incivility, were associated with dehumanisation. These results allowed us to select behaviours that varied in incivility for Study 2. In Study 2 (*N *= 402), we tested the effectiveness of moral disengagement mechanisms employed by the transgressor depending on the level of incivility of the immoral behaviour. To this end, we employed a 3 (moral disengagement mechanisms: moral justification vs. displacement of responsibility vs. distortion of consequences) x 2 (incivility: high vs. low) between-subjects experimental design. The variance analyses showed that using moral justification or displacement of responsibility led to the least amount of dehumanisation, while distortion of consequences resulted in the highest level of dehumanisation. Additionally, immoral behaviours that were high in incivility led to greater dehumanisation than those that were low in incivility, regardless of the moral disengagement mechanism. Overall, our research highlights the significance of different moral disengagement mechanisms and civility as key factors that affect how bystanders perceive the humanness of moral transgressors.

## Introduction

When people engage in unethical or immoral behaviour, they often justify their actions by using moral disengagement mechanisms, attempting to persuade themselves and others that their misbehaviour is morally acceptable [1,2]. These justifications typically involve reinterpreting the behaviour’s meaning by transforming it into an acceptable behaviour, transgressors discharging themselves of responsibility for performing the behaviour, or distorting the consequences of the action [3,4].

Since morality is one of the characteristics of what it means to be human [5,6], these moral disengagement mechanisms should also influence how the transgressor’s humanness is perceived. However, the effectiveness of these moral disengagement mechanisms and their consequent effect on the transgressor’s humanisation are unlikely to be the same for all behaviours. The effectiveness of moral disengagement will depend not only on the severity of the behaviour but also on its implicit characteristics, such as what moral foundation it violates or to what extent it is uncivil.

Thus, in this study, we focus on an implicit characteristic of immoral behaviour: civility. We define civility as the extent to which an immoral behaviour directly impacts satisfactory social relationships. We aim to examine how explanations based on different moral disengagement mechanisms affect the humanisation of the transgressor, depending on the implicit level of civility of the immoral behaviour. We expect that the higher the level of a behaviour’s incivility, the less effective the moral disengagement mechanisms will be to prevent the dehumanisation of the transgressor.

Our contributions are threefold. First, we advance the research on moral disengagement by considering the impact of moral disengagement mechanisms on the extent to which bystanders dehumanise the transgressor. Until now, the literature has shown the efficacy of these mechanisms in reducing internal sanctions [1,7], and promoting more lenient judgements of transgressions by both individuals and ingroups [8,9]. Second, we contribute to the literature on moral foundations [10] by examining how violations of the different moral foundations relate to the transgressor’s perceived humanity. In doing so, we adopt the perspective that a single behaviour may transgress multiple moral foundations simultaneously rather than being confined to a single moral content category [11]. Our study not only test the replicability of previous findings [11] but also expands them by using text-based stimuli. This will also allow us to contribute to the literature by providing a dataset of textual stimuli. Furthermore, no research has examined how each of the moral foundations relate to transgressor’s perceived humanity. Finally, we advance previous research by studying civility as another implicit dimension of immoral behaviour and assess how it impacts the degree to which bystanders humanise the transgressor. Until now, research has examined the relationship between civility and humanity in isolation without integrating civility and moral transgressions. Accordingly, we argue that civility, much like moral foundations, is not a discrete category. Rather, each immoral behaviour may have a higher or lower level of civility [12].

### The impact of moral disengagement mechanisms on moral judgement

People wish to think they are righteous, even though they often commit acts that transgress social norms, and moral disengagement mechanisms are used to restore that desired positive moral identity [7,13]. These mechanisms serve as mental loopholes that people employ to absolve themselves of responsibility and justify their questionable behaviour [3,14]. Furthermore, research reveals that these mechanisms are effective in detaching immoral behaviour from moral standards, thus facilitating immoral actions [1,3]. Moreover, using moral disengagement mechanisms is associated with committing delinquent acts [3,14] and with decreased intentions to implement redistributive justice towards in-group members who commit political violence [15]. Therefore, understanding the effect of these intuitive mechanisms is crucial to fully comprehending moral judgements and attributions of humanness.

It is important, however, to question whether all moral disengagement mechanisms have the same effect on the observer. For instance, if someone is caught being unfaithful to their partner, they may claim various motives for their behaviour, such as: (a) their relationship was already in trouble, and having an affair was the only way to feel alive and reignite passion (moral justification); (b) it was unintentional because they were under the effects of alcohol (displacement of responsibility); or (c) kissing another person for just one night should not be considered infidelity (distortion of consequences). We argue that these moral disengagement mechanisms would have different effects on how observers perceive the severity of the behaviour and the extent to which they attribute humanness to the transgressor.

This idea is based on conceptual differences between the mechanisms [3,14]. Moral justification (behaviour locus) reconstructs harmful behaviour as personally and socially acceptable. By using this mechanism, one acknowledges responsibility for what has happened but considers a higher goal, thus portraying oneself as morally exemplar. Studies have found that people can feel morally righteous even when committing immoral acts against others. For instance, the theory of virtuous violence [16] establishes that violent acts are done to regulate social interactions according to a set of moral rules. In other words, by using a moral disengagement mechanism, people adopt a consequentialist moralism, where the ends justify the (immoral) means. This mechanism individuals to avoid seeing themselves as immoral but instead as morally righteous [17]. Therefore, we consider moral justification as the most powerful mechanism for a transgressor to avoid being dehumanised.

Another option people use to avoid appearing immoral is to lessen the degree to which they are implicated in the action. Through the mechanism of displacing responsibility (agency locus), the individual acknowledges that what has occurred is negative but assigns part or all of the responsibility to external factors. Therefore, they can be spared sanction by stating that they are not the true agents of their actions, as research suggest that the agency of the transgressor is a crucial factor in whether people regard a certain action as immoral [18].

The final mechanism we will examine involves distorting the consequences of the action (outcome locus). In this case, the transgressor aims to demonstrate that the outcome is not harmful, making active efforts to downplay the apparent damage. By minimising or outright denying the adverse results, the transgressor diminishes the observers’ reasons to attribute the action to immorality. Although harm recognition is another key aspect of moral judgements [18], this mechanism may not efficiently restore a positive moral image because observers have already witnessed the harm, and this assessment is resistant to change. Therefore, we expect that this will be the least effective mechanism in eluding dehumanisation.

We have argued that each mechanism will have a different impact. Similarly, we also expect that their efficacy will not be the same when applied to all behaviours but will depend on the implicit characteristics of each immoral behaviour. We will discuss some of the most important of these implicit features below.

### Characteristics of immoral behaviours

Researchers studying moral psychology have made significant strides in developing validated sets of stimuli that capture moral and immoral behaviours. To categorise these behaviours, they have employed various frameworks, including moral foundations [19], traits manifested in behaviours [20,21], various social norms [22], and other moral features [23,24].

Behaviours have been studied through diverse categories [25], with moral foundations theory [26–28] having a particularly profound impact. The theory has been employed as a structured framework to examine various moral content. It categorises social or moral intuitions into distinct and independent ‘foundations’ that are observed cross-culturally and act as a ‘first draft’ of the moral mind [10,28,29]. The core theory identifies five primary foundations: care (concern for others to prevent or alleviate their suffering), fairness (identifying cheating and exploitation), loyalty (engaging in self-sacrifice for group benefit and preventing betrayal), authority (respecting and obeying superiors), and purity (avoiding pathogens – for example, by regulating sexual and eating behaviours). This pluralistic perspective asserts that each of these concerns emerged to address specific social challenges [10].

Moreover, according to moral foundations theory, the moral mind undergoes changes within cultural contexts. Thus, it is possible to study which moral principles are valued or devalued by different groups of people. For example, in the United States, purity, in-group/loyalty, and authority/respect themes tend to be valued more highly by conservatives than by liberals [29,30] and by working-class adults as opposed to university students. These principles are also more highly valued by non-Western populations in general [31,32]. Researchers have used these moral foundations to analyse moral behaviour, leading to the creation of moral stimulus sets in which behaviours are described as disregarding moral norms [11,19].

When people witness moral transgressions, they often dehumanise the transgressor [33]. However, the observer’s evaluation of a behaviour may differ depending on the moral foundation it violates. One of the secondary aims of this paper is to determine whether different types of transgressions differ in terms of how strongly they elicit dehumanisation.

### Civility as a dimension for analysing moral behaviour

Taking our endeavour a step further, we propose civility as another category in the analysis of immoral behaviours, something not contemplated in previous studies. Civility refers to standards of courtesy, good manners, good citizenship, and the well-being of all members of the community [34–36]. Research on civility has predominantly focused on specific contexts. In cities uncivil behaviours are stressors that detrimentally affect the quality of life [36,37]. In organisations, these behaviours relate to leaders’ abuses of authority [38] and the importance of courteous workplace behaviour [39,40]. In education, student incivility disrupts the teaching–learning process [41] and shows disrespect [42]. On social media, uncivil behaviour includes aggressive comments, heated arguments, rude criticisms, sensationalistic claims, hate speech, and harassment [43]. In all these varied settings, however, it is clear that civility is essential for maintaining satisfactory social relationships.

Our understanding of civility encompasses two crucial aspects. Previous research has attempted to study incivility and immorality as if they were two independent types of behaviours, characterising an action as either civil/uncivil or moral/immoral [44]. However, instead of regarding civility and morality as independent, we argue that civility is a non-discrete [12] and cross-sectional aspect of behaviour. This interpretation suggests that varying degrees of incivility can be present within different immoral acts. Accordingly, we aim to examine civility in a wide range of behavioural domains rather than in a single specific one.

### The impact of civility on humanness

Civility is a relevant factor in attributing human features to moral transgressors because the lack of it is intimately related to the dehumanisation of a group or an individual [45–47]. Infrahumanisation theory postulates that people tend to see members of their in-group as more fully human compared to those of the out-group. Infrahumanisation occurs when people associate basic primary emotions (e.g., joy or fear) – which humans share with other animals – equally with the in-group and out-groups, while predominantly attributing positive and negative complex secondary emotions (e.g., hope and guilt) to the in-group [47,48]. According to this theory, these secondary emotions are not universal; they are caused by internal variables, involve high cognition or morality, and are characteristic of human beings [49]. These sophisticated characteristics depend on culture, education, and contact with others, which in turn require behaving in accordance with norms of civility.

The dual model of dehumanisation [46] posits an even more direct connection between civility and humanness. This model defines humanness in two ways: through essential attributes that do not distinguish humans from other creatures (but constitute humans’ natural attributes) or through attributes exclusive to humans. Among the attributes exclusive to humans are civility, refinement, moral sensitivity, rationality, and maturity. When an observer denies these traits to an individual, they apply an animalistic dehumanisation, and the target’s behaviour is perceived as less cognitively mediated than that of others; instead, motives, appetites, and instincts are said to drive it. This form of dehumanisation is similar to infrahumanisation because when it occurs, observers perceive the out-group as being closer to animals. Thus, alongside morality, civility may be an important characteristic when assessing the humanness of a transgressor. In support of this idea, a recent study has shown that acting in an uncivil way promotes animalistic dehumanisation [12].

Since our work asserts that civility/incivility is one of the implicit dimensions of immoral behaviour, we propose that engaging in immoral behaviour that also exhibits high levels of incivility will result in greater dehumanisation compared to immoral behaviour that is low in incivility. We anticipate that this dehumanising effect of incivility will persist even when the transgressor employs moral disengagement mechanisms, except when the mechanism utilised is moral justification. When the transgressor explains that the action was motivated by another moral reason, we expect this mechanism to counteract the dehumanising effect of incivility that other mechanisms are unable to neutralise.

### Overview

We investigate whether all moral disengagement mechanisms equally justify different transgressive behaviours. Specifically, our objective is to determine whether moral disengagement could attenuate the perceived dehumanisation of the transgressor, depending on the degree of incivility of the immoral behaviour. To accomplish this, we undertake two studies.

**Fig 1:**
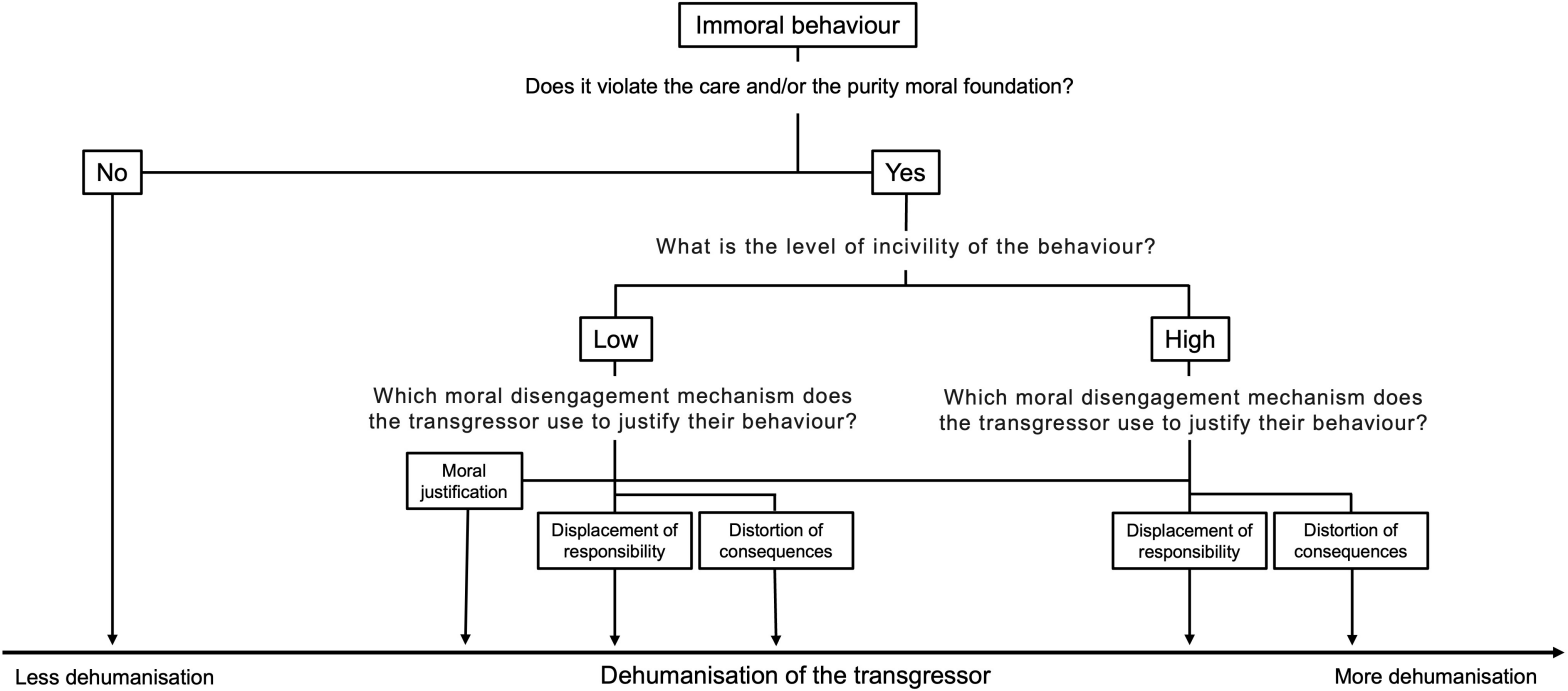
Level of dehumanisation of the transgressor depending on the characteristics of the immoral behaviour and moral disengagement mechanisms.

In the first study, we will analyse different behaviours based on the moral foundations they violate, their degree of incivility, and their capacity to cause observers to dehumanise the transgressor. Using this approach, we will obtain a panel of behaviours that vary based on these dimensions. In a second, preregistered experimental study, we will investigate the effectiveness of different moral disengagement mechanisms on preventing the dehumanisation of the transgressor. Our main goal is to determine how moral disengagement mechanisms impact the dehumanisation of the transgressor depending on the level of incivility of the immoral behaviour. In Fig 1, we present a conceptual framework of our research question.

## Study 1

In this study, we analyse immoral behaviour under the premise that different immoral behaviours may be simultaneously judged based on different dimensions, following the approach and findings of Crone *et al.* [11].

Previous research categorising behaviours based on moral foundations theory [28] has assumed that each behaviour represents a violation of a specific set of values. One notable example of this approach is the study by Clifford *et al.* [19]. These authors asked participants to classify a large number of scenarios according to the moral foundation they violated. Additionally, participants could indicate that behaviours violated social norms that are not considered moral norms. Participants had to choose a single motive, disregarding the possibility that an action could simultaneously violate multiple norms. Later, Crone *et al.* [11] tested an alternative approach and showed that the transgressor in each scenario may simultaneously violate more than one moral foundation rather than having a primary association with a single one.

Building on the latter approach, we assess how individuals rate different behaviours on all moral foundations while additionally incorporating the dimension of civility. We expect that different behaviours may violate multiple dimensions simultaneously, rather than being exclusively ascribed to one of them. In addition, we aim to examine how the different moral foundations and civility attributed to each behaviour relate to dehumanisation. We predict that behaviours related to incivility will prompt a greater degree of dehumanisation than those not linked to this dimension. Although we expect behaviours that transgress moral foundations to be associated with dehumanisation, we have no a priori hypotheses regarding the differential effects of the various moral foundations on levels of humanness.

### Method

#### Ethical statement.

The studies received approval from the Ethics Committee on Research and Animal Welfare of University of La Laguna for research involving human participants. Prior to participating, all individuals were required to review the consent form, voluntarily agree to take part, and provide written informed consent.

#### Participants.

To maximise our available resources, we followed an effect-size sensitivity procedure for sample size determination. This procedure allows us to recruit all possible participants and assess the power of the resulting analyses. The method is recommended by Open Science Framework [50]. A total of 326 Spanish individuals completed an online survey hosted on Qualtrics. Participants were either recruited via the online platform Prolific [51] and received a financial reward of £1,45, or they were psychology students from University of La Laguna and received course credits. After excluding those who failed the attention check questions, we obtained a final sample of 302 participants (*M*_*age *_= 28.09, *SD* = 11.14, 49.30% women). Of the participants, 174 were recruited from Prolific and 128 were psychology students. The education levels were 4.60% primary or secondary education, 25.65% higher vocational training or high school, and 69.75% university studies. A sample of 200 participants is suggested for conducting bivariate correlations [52], and a sensitivity analysis (G*Power software [53]) confirmed that for a power of 0.90, we could detect a correlation as small as *r *= .184.

#### Material: Stimuli.

We examined a total of 61 behaviours. We focused on behaviours that transgressed one of the moral foundations (care, fairness, loyalty, authority, purity) or civility norms, and we also included two nondeviant behaviours. According to our theoretical approach, each immoral behaviour could simultaneously transgress several moral foundations and civility standards. However, the 61 behaviours elaborated as stimuli were devised as paradigmatic examples of violations to either one of the foundations or one of the civility norms, as per previous normative studies [19–21]. In other words, each stimulus prompt participants to identify the violation of a single foundation or civility norm. To construct the scenarios for each moral foundation, we mainly employed original or modified forms of the moral foundations vignettes by Clifford *et al.* [19] or adapted related, specialised research [12,20–22,54]. Stimuli adapted from studies in English were translated by using a back-translation process to ensure linguistic accuracy. Additionally, all stimuli were reviewed by experts from two research groups from University of La Laguna that specialise in dehumanisation. Our selection process gave precedence to actions that would be familiar to a broad range of individuals, either through personal experience or through direct or indirect observations.

We sought to emphasise the transgressive behaviour itself and minimise the influence of other variables on moral judgements. Therefore, the vignettes exclusively described actions (i.e., cheating) without specifying other potentially biasing information, such as the gender or ethnic identities of characters. In an attempt to minimise attrition, we ensured that the scenario descriptions were highly concise (ranging from 4–19 words or 18–66 characters), and gender-neutral whenever feasible. Below, we specify the shared characteristics of each group of vignettes referring to a specific violation of moral foundations or civility.

For the care foundation, we selected eight behaviours that involved either emotional harm to a human, physical harm to a human, or physical harm to a nonhuman animal (e.g., ‘harassing someone on social media’, ‘spanking your child for getting bad grades in school’).

We selected seven behaviours that violated the fairness foundation but avoided scenarios related to ethnicity, gender, or structural inequity. Some examples include ‘cheating at a board game’, and ‘calling in sick when you are feeling well to avoid going to work’).

For the loyalty foundation, we chose eight behaviours in which an individual puts their interests before those of family, friends, or workplace groups, among others (e.g., ‘telling family secrets when family members have asked you not to do so’, ‘secretly dating your best friend’s crush’).

For the authority foundation, the nine behaviours involve disobedience or disrespect towards traditional authority figures and institutions or symbols of authority, such as parents, teachers, coaches, bosses or police. Examples include ‘ignoring the curfew set by parents’ and ‘replying to the police in another language so they cannot understand you’.

For the purity foundation, we selected eight behaviours that trigger disgust, including sexually deviant behaviours (e.g., ‘rubbing up sexually against a stranger on public transport’) and behaviours that in some way degrade one’s body (e.g., ‘wearing the same underwear for two weeks’).

Regarding civility, we selected 21 behaviours that either involve physically mistreating shared spaces (e.g., ‘putting rubbish outside the bin’, ‘damaging street furniture’) or more direct interaction with other people (e.g., ‘interrupting someone when they are speaking’, ‘skipping turns in a queue’).

Finally, we included two valence-neutral behaviours that should not break any social norms. These were: ‘putting a letter in the mailbox’ and ‘turning the pages of a book’.

##### Measures.

*Moral foundations and civility*. To measure the extent to which each behaviour was associated with violating each moral foundation and transgressing civility, we presented participants with a series of items to rate on a 1–7 scale. We assessed each moral foundation, as well as civility, using a single item. We included five items, each of which corresponded to a moral foundation (e.g., ‘this action is immoral because it is harmful and cruel and causes suffering’ [care], ‘this action is immoral because it defiles one’s body and degrades it’ [purity]). We also included one item related to uncivil behaviour (e.g., ‘this action is uncivil because it lacks courtesy and good citizenship’).

*Dehumanisation*. We used an explicit dehumanisation scale called the ascent of human scale (AOH). Before presenting the scale, we asked participants to read the following instruction: ‘Although we are all human beings, some people do things that make them seem highly evolved, while others, because of what they do, seem indistinguishable from animals. Using the image below as a guide, indicate on a scale of 0 to 100 how evolved you think the people who perform the following behaviours are, with 0 being less “evolved” and 100 more “evolved”.’ Subsequently, participants viewed the images developed by Kteily *et al.* [55], followed by a slide that prompted them to assign their score.

*Incorrectness of the behaviour*. To assess the participants’ incorrectness valuation of the different behaviours, we used a scale composed of three items. The first item was *inappropriateness of the behaviour*, which read: ‘Indicate from 1 to 7 the extent to which these behaviours seem inappropriate to you, with 1 being “not inappropriate” and 7 being “fully inappropriate”.’ The second item was *severity of the behaviour*, which read: ‘Please indicate from 1 to 7 the extent to which you consider it severe that people carry out these types of behaviours. Remember that 1 is “not severe” and 7 is “fully severe”.’ The last item was *intention to punish the behaviour*, which asked: ‘To what extent do you believe that this behaviour should be punished? Remember that 1 is “it does not deserve punishment” and 7 is “it deserves a lot of punishment”.’ The internal consistency for the scale proved to be excellent in the current sample (α = .92).

##### Procedure.

After providing informed written consent, participants were directed to the online questionnaire. To prevent fatigue to the participants, we created eight versions of the questionnaire. Each contained at least one behaviour from each moral foundation, two or three behaviours that were uncivil, and one neutral behaviour. Approximately 40 individuals rated each action. Each participant was randomly assigned to one out of eight versions of the questionnaire and asked to respond to it, rating 8–9 behaviours. Participants assessed each behaviour with regard to all dependent variables: the degree to which they violated each moral foundation, the degree to which they were uncivil, the amount of dehumanisation and the perceived incorrectness of the behaviour.

##### Data analysis.

We calculated the mean scores for all the stimuli on all dependent variables. Then, to merge the responses of all questionnaires, we transferred the results to a new matrix. The new matrix was transposed so that the behaviours were located in the column where the participants’ scores normally appear. This analytical approach replicated similar protocols used in previous related research [56,57]. Next, we examined the descriptive scores of all dependent variables for each behaviour. Finally, we conducted bivariate correlation analyses for each of the moral foundations and for civility, as well as for the dependent variables of dehumanisation of the transgressor and incorrectness of the behaviour. We set a significance level of.05 for these analyses. We used the IBM SPSS Statistics 29, a software that provides a wide range of statistical tools and delivers highly accurate results, making it a staple of inferential statistics in psychology for decades [58].

### Results

#### Moral foundations and incivility.

We examined the descriptive results regarding the reason each behaviour was considered inappropriate. Our findings revealed that each behaviour could transgress multiple norms within the domain of immorality, while also displaying varying levels of incivility (see S1 Appendix).

To assess the relationship between different moral foundations, and between these foundations and incivility, we conducted several bivariate correlation analyses. As Table 1 shows, perceived violations of care correlated positively and significantly with perceived violations of fairness (*r* (60) =.610, *p *< .001), loyalty (*r* (60) =.650, *p *< .001) and purity (*r* (60) =.475, *p *< .001). We used Fisher’s *r*-to-*z* transformations to compare these correlation coefficients without finding significant differences between them (*p*s ≥ .09). In addition, perceived violations of fairness correlated positively with perceived violations of loyalty (*r* (60) =.680, *p *< .001). Finally, perceived violations of fairness (*r* (60) =.349, *p *= .006) and perceived violations of authority correlated positively with incivility (*r* (60) =.330, *p *= .01).

**Table 1:** Intercorrelations between the five moral foundations and incivility.

	C/H	F/C	L/B	A/S	P/D
Care/Harm	–				
Fairness/Cheating	.610**	–			
Loyalty/Betrayal	.650**	.680**	–		
Authority/Subversion	.128	.242	.180	–	
Purity/Degradation	.475**	.058	.023	-.037	–
Incivility	.203	.349*	-.065	.330*	-.140

*Note.* **p < .001, *p < .01.

#### Dehumanisation.

We performed a bivariate correlation analysis of the moral foundations and incivility scores with the dehumanisation score. We expected behaviours related to incivility to correlate with dehumanisation. We did not have a specific hypothesis regarding the differential effect of dehumanisation across the various moral foundations. On this scale, a low score shows greater dehumanisation than a high measure because a measure close to 0 means that the agent is ‘less evolved’ than a score close to 100, where the agent would be ‘more evolved’. Our analysis showed that the relationship between dehumanisation and the moral foundation scores was only significant in the care/harm and purity/degradation dimensions (see Table 2).

**Table 2:** Intercorrelations between the different types of transgressions and their associated dehumanisation and incorrectness.

	Dehumanisation	Incorrectness
Care/Harm	−.526*	.752*
Fairness/Cheating	−.242	.605*
Loyalty/Betrayal	−.039	.477*
Authority/Subversion	−.109	.429*
Purity/Degradation	−.679*	.546*
Incivility	−.421*	.471*

*Note.* *p < .001.

Specifically, the data showed that the more harm a behaviour exerted on others or the more it degraded the body, the less humanness the observer attributed to the transgressor. Additionally, dehumanisation correlated significantly with incivility (*r* (60) = − .421), showing that the higher the incivility score, the more dehumanisation occurred. However, the analysis of the differences between the correlations showed that the relationship between purity/degradation and dehumanisation was significantly stronger than the relationship of care/harm (*z* = 1.81, *p* = .035) and incivility (*z* = 2.41, *p* = .008) with dehumanisation. No differences were noted between the association of care/harm and incivility with dehumanisation (*z* = 0.731, *p* = .23).

#### Incorrectness of the behaviour.

We were also interested in determining whether behaviours that transgressed each of the moral foundations or norms of incivility were considered to be equally incorrect. According to the results, all behaviours positively correlated with the incorrectness rating of the act (see Table 2). We examined the differences between correlations with Fisher *z* statistics. The results showed that the correlation between incorrectness and care/harm was significantly stronger than the correlations between incorrectness and other moral foundations or incivility (*z*s > 1.65, *p*s < .048).

### Discussion

Our main objective in this study was to examine the moral content of immoral behaviours. Specifically, we aimed to assess whether these behaviours could be appraised as simultaneously violating several moral foundations and civility norms, as well as the extent to which these judgements were associated with dehumanisation.

Our findings indicate that most of the behaviours examined in our study can simultaneously violate multiple moral categories. This conclusion aligns with previous research on mixed moral content, which used images to demonstrate that a single stimulus could map onto more than one moral domain [11]. Our study expands this approach by employing text-based stimuli instead of images.

We also find that moral foundations differ in their association with dehumanisation. Notably, inflicting harm or disregarding body purity correlated significantly with dehumanisation, whereas other moral foundations did not. Regarding the care foundation, previous theories have posited that preventing harm is, in fact, the primary function of morality, and that harm serves as the main criterion for determining whether an act is immoral [59,60]. Meanwhile, purity violations are perceived as ‘weird’ [61], ‘disgusting’ [62], and ‘uncommon’ acts [63] that are attributed to a person’s poor character and have been observed to lead to social isolation for the transgressor [64]. Thus, not only is the act itself perceived as wrong, but it also implies something negative about an individual’s moral character. Consistent with this idea, our results indicate that to be considered a fully human being, people must adhere to the moral foundations of care and purity. In contrast, immoral behaviours that transgress other foundations may be perceived as negative, but they do not seem to have a significant effect on the extent to which the transgressor is dehumanised. By highlighting this link between care and purity moral foundations and the dehumanisation of moral transgressors, our research contributes to the study of morality in general and moral foundations in particular.

Additionally, incivility varied independently of the attributed morality of each stimulus, supporting our assumption that civility is an implicit characteristic of moral transgressions. Furthermore, it was also associated with dehumanisation. Thus, observers attribute less humanness to those who violate civility, a finding that aligns with previous research demonstrating that incivility promotes animalistic dehumanisation [12,65].

In summary, this study provides a foundation for Study 2. The results indicate that immoral behaviours can violate more than one norm at a time [11] and that civility plays a role in how observers attribute humanness to transgressors. Moreover, the study provides a set of pretested behaviours that can be confidently used for our manipulations in Study 2. In Study 2, we build on these findings to examine whether moral disengagement mechanisms mitigate the dehumanising effect of care and purity violations, while also considering the impact of incivility.

## Study 2

In this study, we investigate how different moral disengagement mechanisms (including moral justification, distortion of consequences, and displacement of responsibility) mitigate the extent to which a transgressor is dehumanised, depending on the level of incivility of the immoral behaviour. To this end, we focus on behaviours that mainly transgress care or purity (the two moral foundations associated with dehumanisation, according to the results of Study 1) and that may be high or low in incivility while keeping a consistent level of severity. Overall, we anticipate that the impact of moral disengagement on the perception of the transgressor’s humanness will vary depending on the transgression’s level of incivility.

First, we aim to examine the impact of moral disengagement mechanisms. Our initial hypotheses posit that, based on conceptual differences between the mechanisms [3], certain mechanisms will lead to higher levels of dehumanisation than others. From a consequentialist perspective, where the ends justify the means, an immoral act can be seen as morally justified if it is performed to defend one’s moral values [16,17]. Based on this theoretical background, we predict that moral justification will be the most effective mechanism for preserving or enhancing the transgressor’s humanness, as it frames the act as morally virtuous and thus portrays the transgressor as a moral individual. In contrast, we anticipate that distorting the consequences of the act will be the least effective mechanism in reducing dehumanisation. Recognising harm is a crucial element in making moral judgments [18], and once harm has been acknowledged, it might be more resistant to reinterpretation. Moreover, this mechanism does not attempt to influence any other element of the moral judgement scenario (i.e., the intention of the transgressor or vulnerability of the victim). Finally, we predict that displacement of responsibility will fall in between: While the transgressor employing this mechanism recognises the harm, they claim it occurred unintentionally [18]. Although this may mitigate dehumanisation to some extent, we predict it will not do so as effectively as moral justification, which ultimately paints the transgressor in a positive light.

Hypothesis 1: When the transgressor employs the distortion of consequences mechanism, they will be dehumanised to a greater extent than when using other moral disengagement mechanisms.

Hypothesis 2: When the transgressor employs the moral justification mechanism, they will be dehumanised to a lesser extent than when using other moral disengagement mechanisms.

Next, we aim to test whether a higher degree of incivility causes increased dehumanisation of the transgressor. The rationale for this hypothesis comes from the idea that civility is an attribute unique to humans [46], which is closely linked to sophistication, culture [48] and, therefore, social interaction. Thus, an individual performing a behaviour that is high in incivility would be perceived as lacking such an essential human characteristic and, consequently, dehumanised [12]. Much like the theoretical framework provided by the literature, our own empirical data supports this hypothesis, as Study 1 revealed an association between incivility and dehumanisation. In the present study, we further test the relationship between these factors by assessing causality. Specifically, we examine whether manipulating the civility of an action influences the degree of dehumanisation directed towards the transgressor.

Hypothesis 3: When a transgressor performs a behaviour with a high degree of incivility, they will be dehumanised to a greater extent than when performing a behaviour with a low degree of incivility.

Furthermore, we expect that one of the mechanisms – specifically, moral justification – will nullify the effect of incivility. In moral justification, the transgressor acknowledges the action as wrong but frames it as the best choice due to righteous intentions [3], making the incivility of the action and the inconvenience it causes necessary to protect the greater good. This rationale is absent in the other mechanisms, where incivility is not justified. Thus, we suspect that moral justification is the only mechanism that may neutralise the dehumanising effect of incivility.

Hypothesis 4: There will be no difference in how much a transgressor is dehumanised when they use moral justification to explain a behaviour, regardless of the degree of incivility.

Lastly, we anticipate that the moral disengagement mechanisms will not only alter the effect of dehumanisation but will also impact the level of perceived severity of the behaviours. Specifically, because the intentionality of the transgressor plays a crucial role in how observers judge whether an act is considered wrong [18], we predict that the most effective mechanism for minimising the perceived severity of an action will be the only one in which the transgressor denies responsibility for the behaviour. The other two mechanisms, however, imply that the transgressor had alternatives but chose to act in a particular way.

Hypothesis 5: When the transgressor employs the displacement of responsibility mechanism, the behaviour will be perceived as less severe than when other moral disengagement mechanisms are used.

### Method

#### Design.

To test our hypotheses, we conducted a 3 x 2 between-subjects experiment. The manipulated variables were moral disengagement (moral justification vs. displacement of responsibility vs. distortion of consequences) and incivility (high vs. low).

#### Participants.

Spanish participants were recruited through Prolific, an online recruitment platform that ensures high-quality data and fair compensation to participants [51]. The survey was hosted online on Qualtrics, and all participants gave their written informed consent. The ideal sample size was 431, determined using G*Power software [53], for a power of 0.95, α = .05 and a small to medium effect size (f = 0.19) for the F tests (ANOVA: fixed effects, special, main effects, and interactions). The expected effect size corresponds to the average effect size found on the corresponding area of intergroup relations [66]. To allow for a 25% margin of error in the manipulation and attention check questions, we recruited 541 participants. After excluding 118 participants who failed to identify the moral disengagement mechanism as expected and 12 who failed the attention checks, the final sample included 412 participants (*M*_*age*_* *= 28.35, *SD*_*age*_* *= 9.5, 54.9% women). The education levels were 3.15% primary or secondary education, 24.75% higher vocational training or high school, and 71.60% university studies.

#### Material: Stimuli.

*Immoral behaviours*. Immoral behaviours were selected for this study based on the findings of Study 1. We specifically chose four behaviours that strongly violated moral norms (two against purity norms – ‘sexually rubbing against a stranger on public transport’ and ‘vomiting in the middle of a meal in order to continue eating’ – and two transgressing care norms – ‘harassing someone on social media’ and ‘spanking your child for getting bad grades in school’).

We chose these behaviours because of the correlations we observed between these moral foundations and the dehumanisation of the transgressor (*r* (60) _Purity_ = −.677, *r* (60) _Care_ = −.526, *p*s < .001). This pattern extends beyond these specific norm violations, as transgressions of civility norms also exhibit a similar correlation with dehumanisation (*r* (60) _Incivility_ = −.421, *p* = .001). Thus, in line with the findings of Study 1, we selected two immoral behaviours that were related to these foundations but differed in their degree of incivility. The selected behaviours also maintained the same level of perceived severity. Our hypotheses did not seek to differentiate between the moral foundations of purity and care. Rather, we incorporated both types of moral norms associated with dehumanisation to ensure a more complete representation of stimuli.

Regarding the behaviours that violated purity norms, no significant differences were found concerning the extent to which people consider these behaviours severe (*t* (74) = 1.41; *p* = .019; *d* = 0.23, 95% CI [−0.095, 0.556]). However, the behaviours differed in civility (*t* (74) = 4.49; *p* < .001; *d* = 1.88, 95% CI [1.047, 2.720]). We observed a similar pattern for the two behaviours that violated the standard of care. Both scored similarly in severity (*t* (72) = −1.62; *p* = .109; *d* = −0.23, 95% CI [−0.512, 0.053]) but differed significantly in civility (*t* (72) = 3.44; *p* < .001; *d* = 1.62, 95% CI [0.683, 2.563]). Thus, both behaviours equally transgress the purity or care moral foundations and are perceived as equally severe, but one is considered less civil than the other.

*Moral disengagement*. The description of each of these behaviours was followed by a form of moral disengagement, derived from individual interviews and expert group sessions. Specifically, (a) four arguments provided moral justifications for each behaviour (e.g., in the story of rubbing on public transport: ‘There were a lot of people on the underground, and I saw a person try to open her backpack. So, it occurred to me to stick very close to her to prevent her from being robbed’); (b) four arguments attempted to displace responsibility from the agent (e.g., ‘I brushed against her because the underground was very full, the train braked and I bumped into her a lot, but I didn’t mean to’); and finally, (c) four arguments distorted the consequences of the action (e.g., ‘I brushed against her, but she barely noticed, and I don’t think she remembers. It’s something that happens all the time, and I don’t think it’s such a big deal’).

To confirm that the participants classified these arguments into three categories, they were asked after reading the vignette to indicate which of the following expressions best described the protagonist’s explanation. Specifically, they were given three options: ‘They did it for the greater good, seeking the welfare of another’ (moral justification), ‘It happened unintentionally and because an unexpected event occurred’ (displacement of responsibility), and ‘They did it thinking that the action had no negative consequences’ (distortion of consequences). The chi-square analysis conducted with the hits and misses in the six experimental conditions resulted in χ2_(4)_ = 514.889 (*p* <.001), indicating a relationship between the participants’ responses and the expressions the transgressors used to justify their actions. The analyses in this study were only conducted with participants who correctly categorised these expressions (*N* = 412, 76.16%).

**Measures**.

*Dehumanisation*. We used two different measures of dehumanisation: blatant and subtle. As an explicit measure, we employed the same scale utilised in Study 1, the AOH [55], and followed an identical procedural approach. In addition to this measure, we also incorporated the infrahumanisation scale [48], which captures implicit tendencies and assesses the attribution of primary and secondary emotions. In this case, the participants were instructed to reflect upon the individuals involved in the behaviours and were then asked: ‘To what extent do you think that in their daily life they may experience the following?’ Using a 7-point scale ranging from 1 (*not at all*) to 7 (*completely*), participants indicated the transgressor’s capacity to experience emotions. The scale encompassed six primary emotions (excitement, joy, pleasure, fear, sadness, and anger) and six secondary emotions (friendship, compassion, hope, guilt, remorse, and shame). The internal consistency for the entire scale proved to be excellent in the current sample (α = .884), as well as the subset focusing solely on the secondary emotions (α = .891). The emotional terms were selected from a normative study [67], which ensured they were effectively different in terms of humanness but had the same valence. Primary emotions exhibited lower levels of humanness (*M* = 2.55, *SD* = 0.47) compared to secondary emotions (*M* = 5.39, *SD* = 0.73; *t*(10) = 8.03, *p *< 0.001) on a scale ranging from 1 (shared by animals and humans) to 7 (exclusively human). However, both primary and secondary emotions demonstrated similar levels of valence (*M* = 3.94, *SD* = 2.50 for primary emotions; *M* = 3.44, *SD* = 2.10 for secondary emotions; *t* (10) = 0.37, *p* = .716) on a scale from 1 (very unpleasant) to 7 (very pleasant).

*Severity of the behaviour*. Although we selected behaviours of equal severity based on the results of Study 1, we still assessed it in this study (‘To what extent do you consider what this person has done to be severe?; 1: not severe; 7: very severe)’ to analyse whether pairing the behaviour with different moral disengagement mechanisms can indeed modify the perceived this variable.

#### Procedure.

At the beginning of the questionnaire, the participants were informed of their rights and asked for their written consent. Then, they read a brief passage explaining how people regularly witness and judge the daily behaviours performed by others. Participants were informed that they would be presented with a description of a specific behaviour that occurred, along with an explanation provided by the individual responsible, referred to as MH. They were instructed to read the information carefully, reflect upon it, and respond to the subsequent questions honestly. The following is an example of one of the behaviour descriptions: MH sexually brushed against a person on the underground, and someone who saw what was happening reproached him for his action. MH said, ‘There were a lot of people on the underground, and I saw a person try to open her backpack. So it occurred to me to stick very close to her to prevent her from being robbed’. After reading the vignette, the participants were directed to respond to the manipulation check. Subsequently, the remaining dependent variables of dehumanisation and severity appeared, along with sociodemographic variables.

#### Data analysis.

To test our hypotheses, we conducted three separate analyses of variance (ANOVAs), one for each dependent variable (AOH, infrahumanisation, severity of the behaviour). In all analyses, moral disengagement mechanisms and incivility were included as factors. We set a significance threshold of .05. We used the IBM SPSS Statistics 29 software due to its reliability in inferential statistics in psychology [58].

### Results

To test Hypothesis 1, we examined the impact of moral disengagement on both AOH and infrahumanisation. First, since the results showed a significant main effect of the moral disengagement variable on AOH (*F*(1,411) = 5.51, *p* = .004, η_p_^2^ = .026), we conducted a pairwise comparison analysis with a Bonferroni adjustment. The results supported Hypothesis 1, indicating that participants attributed the lowest level of humanness to the transgressor when they used the distortion of consequences mechanism (*M* = 45.97, *SD* = 25.73). This level of humanness was significantly lower than that elicited when the transgressor employed moral justification (*M* = 54.48; *SD* = 27.63; *p* = .025) and displacement of responsibility (*M* = 56.03, *SD* = 29.25, *p* = .008).

Likewise, in line with Hypothesis 1, the analysis of the infrahumanisation responses revealed that the moral disengagement variable had a significant effect (*F* (1,411) = 13.07, *p* < .001, η_p_^2^ = .061). The Bonferroni pairwise comparison analyses showed that when the transgressor employed the distortion of consequences mechanism, they received the highest levels of infrahumanisation. Participants attributed the lowest number of secondary emotions to transgressors who used this mechanism (*M* = 3.22, *SD* = 1.29), significantly lower than for those employing moral justification (*M *= 3.74, *SD *= 1.47, *p* = .004) and displacement of responsibility (*M *= 4.09, *SD *= 1.64, *p *< .001).

In Hypothesis 2, we anticipated that moral justification would be associated with higher levels of humanness compared to displacement of responsibility and distortion of consequences. However, the analysis of the response to the AOH scale revealed no significant differences between the mechanisms of moral justification (*M* = 54.48, *SD* = 27.63) and displacement of responsibility (*M* = 56.03, *SD* = 29.25; *p* = 1.000). Furthermore, the results from the infrahumanisation scale revealed no difference between the mechanisms of moral justification (*M *= 3.74, *SD *= 1.47) and displacement of responsibility (*M *= 4.09, *SD *= 1.64, *p* = .169). Therefore, although moral justification resulted in some of the lowest levels of dehumanisation, we were unable to fully verify Hypothesis 2.

To test Hypothesis 3, we examined the impact of incivility on both AOH and infrahumanisation. The results for AOH revealed that incivility had a significant effect (*F* (1,411) = 14.84, *p* < .001, η_p_^2 ^= .035). This finding indicated that the participants regarded transgressors of high incivility behaviours as less human (*M *= 47.14, *SD *= 29.04) compared to those with low incivility behaviours (*M *= 57.10, *SD *= 25.51). Similarly, the analysis of infrahumanisation yielded consistent findings. Specifically, secondary emotions had a significant main effect (*F* (1,411) = 43.80, *p* < .001, η_p_^2 ^= .097). Participants attributed fewer secondary emotions when the behaviour had a high level of incivility (*M *= 3.22, *SD *= 1.49) than when the behaviour had a low level of incivility (*M *= 4.14, *SD *= 1.38).

In Hypothesis 4, we expected that moral justification would produce similar levels of dehumanisation for behaviours with both high and low degrees of incivility. To assess this, we first checked the analyses of double interactions between the variables in AOH. There was no significant interaction between incivility and moral disengagement (*F* (2,411) = 2.18, *p* = .113, η_p_^2^ = .011). However, the Bonferroni post hoc adjustment pairwise comparisons suggested no significant differences in the effect of the moral justification mechanism between the high and low incivility conditions (*p* = .45). In contrast, significant differences were observed for the displacement of responsibility (*p* < .001) and distortion of consequences (*p* = .024) mechanisms. Transgressors in the high incivility condition were perceived as less human than those engaging in less uncivil behaviours (see Table 3).

**Table 3:** Means and standard deviations (in brackets) of Ascent of Human (AOH) measures as a function of experimental conditions.

	Immoral behaviours	
	High incivility behaviour	Low incivility behaviour
Moral justification	52.85 (28.70)ax	56.26 (26.51)ax
Displacement of responsibility	47.49 (32.01)ax	64.71 (23.38)ay
Distortion of consequences	40.98 (25.64)bx	51.19 (24.96)ay

*Note*. Within each row, means that do not a share subscript x, y differ (*p* < 0.05). Within each column, means that do not share a subscript a, b differ (*p* < 0.05). Lower scores indicate stronger dehumanisation.

Next, we conducted independent *t* tests. The results revealed that there were no significant differences in the effect of the moral justification mechanism based on the behaviour’s level of incivility (*t* (142) = 0.738, *p* = .462), in line with Hypothesis 4. However, significant differences were present when the transgressor used the displacement of responsibility (*t* (123) = 3.429, *p* = .001, *d* = .613, 95% CI [7.28, 27.16]) and the distortion of consequences mechanisms (*t* (141) = 2.409, *p* = .017, *d* = .403, 95% CI [1.82, 18.57]).

Finally, we checked for interactions between the independent variables on infrahumanisation responses. The interaction between incivility and moral disengagement was also not significant (*F* (2,411) = 0.064, *p* = .938, η_p_^2^ = .000). We found that all three moral disengagement mechanisms consistently led participants to attribute lower scores of humanness when the behaviour exhibited high incivility compared to low incivility conditions (*p*s < .001). In terms of the implicit measure, none of the mechanisms succeeded in negating the impact of incivility (see Table 4).

**Table 4:** Means and standard deviations (in brackets) of infrahumanisation (attribution of secondary emotions) as a function of experimental conditions.

	Immoral behaviours	
	High Incivility behaviour	Low Incivility behaviour
Moral justification	3.29 (1.45)ax	4.22 (1.34)ay
Displacement of responsibility	3.66 (1.81)ax	4.51 (1.32)ay
Distortion of consequences	2.74 (1.01)bx	3.71 (1.36)by

*Note*. Within each row, means that do not share a subscript x, y differ (*p* < 0.05). Within each column, means that do not share a subscript a, b differ (*p* < 0.05). Lower scores indicate stronger dehumanisation.

To test Hypothesis 5, we conducted an ANOVA on the severity measure. The effect of the moral disengagement variable was statistically significant (*F* (1,411) = 15.94, *p* < .001, η_p_^2 ^= .073). Specifically, in accordance with our expectations, pairwise comparisons with the Bonferroni adjustment indicated that when the transgressor used the displacement of responsibility mechanism (*M* = 4.80, *SD* = 1.79, *p*s* *< .001), the perceived level of severity was lower in contrast to moral justification (*M* = 5.60, *SD* = 1.26) and distortion of consequences (*M* = 5.72, *SD *= 1.27, *p *= 1.000). The latter mechanisms were associated with a higher perception of the severity of the behaviour.

Although we did not consider this analysis in our hypotheses or preregistered study, we also explored whether incivility was related to perceived severity of a behaviour in the same direction as dehumanisation. We first analysed the main effect of incivility on severity and then the interaction between incivility and moral disengagement (see Table 5).

**Table 5:** Means and standard deviations (in brackets) of severity of the behaviour as a function of experimental conditions.

	Immoral behaviour	
	High incivility behaviour	Low incivility behaviour
Moral justification	5.57 (1.10)ax	5.61 (1.41)ax
Displacement of responsibility	5.17 (1.78)bx	4.42 (1.72)by
Distortion of consequences	5.99 (1.11)ax	5.44 (1.37)ay

*Note*. Within each row, means that do not share a subscript x, y differ (*p* < 0.05). Within each column, means that do not share a subscript a, b differ (*p* < 0.05).

The results showed that incivility had a significant effect (*F* (1,411) = 8.94, *p* = .003, η_p_^2 ^= .022), indicating that participants considered immoral behaviours that are high in incivility as more severe (*M *= 5.60, *SD *= 1.37) than those that are low in incivility (*M *= 5.18, *SD *= 1.58). We also observed a significant trend in the interaction between incivility and moral disengagement (*F* (2,411) = 2.82, *p* = .06, η_p_^2^ = .014). Therefore, we conducted post hoc pairwise comparisons using the Bonferroni adjustment. The results did not indicate significant differences in the effect of the moral justification mechanism when comparing behaviours with high and low incivility (*p* = .88). However, significant differences were observed when comparing the level of incivility in the displacement of responsibility (*p* = .003) and distortion of consequences (*p* = .023) conditions, indicating that participants attributed higher severity to behaviours high in incivility compared to those low in incivility when transgressors used these mechanisms. To validate these results, we performed independent *t* tests. The results revealed no significant differences in the effect of the moral justification mechanism based on the level of incivility (*t* (142) =.168, *p* = .867). However, significant differences were found in the displacement of responsibility mechanism (*t* (123) = 2.41, *p* = .018, *d* = .431, 95% CI [0.134, 1.376]) and the distortion of consequences mechanism (*t* (141) = 2.60, *p* = .010, *d* = .435, 95% CI [0.130, 0.957]).

To provide a clearer overview of the findings, in Table 6 we summarize the hypotheses tested in Study 2 and indicate which ones were supported by the data.

**Table 6:** Summary of hypotheses.

Hypotheses	Results
*H*1: When the transgressor employs the distortion of consequences mechanism, they will be dehumanised to a greater extent than when using other moral disengagement mechanisms.	Supported
*H2*: When the transgressor employs the moral justification mechanism, they will be dehumanised to a lesser extent than when using other moral disengagement mechanisms.	Partially supported
*H3*: When a transgressor performs a behaviour with a high degree of incivility, they will be dehumanised to a greater extent than when performing a behaviour with a low degree of incivility.	Supported
*H4*: There will be no difference in how much a transgressor is dehumanised when they use moral justification to explain a behaviour, regardless of the degree of incivility.	Supported in the blatant measure and not supported in the subtle measure
*H5*: When the transgressor employs the displacement of responsibility mechanism, the behaviour will be perceived as less severe than when other moral disengagement mechanisms are used.	Supported

### Discussion

In Study 2, the first objective was to investigate whether different levels of incivility produce different degrees of dehumanisation. We selected four behaviours that transgress to a greater extent the two moral foundations (purity and care) that were most associated with dehumanisation in Study 1. Within each of the moral foundations, we selected one behaviour that exhibited a higher level of incivility and one that exhibited a lower level, while keeping the perceived severity of the behaviour constant. We anticipated that behaviours with higher levels of incivility would lead to greater dehumanisation compared to those with lower levels of incivility. The results demonstrated that the behaviours that transgressed purity and care, and additionally had higher levels of incivility, produced more dehumanisation than behaviours that transgressed the same moral foundations but with lower levels of incivility. This result, consistent with Study 1, confirms that incivility is an important characteristic of immoral acts. It can vary within similarly severe actions and influence the degree of dehumanisation attributed to the transgressor. Ultimately, our research draws the novel conclusion that an immoral act that is also uncivil causes observers to ascribe higher levels of dehumanisation to the transgressor than they would in the case of immoral actions considered less uncivil.

Additionally, our study investigated whether different moral disengagement mechanisms could mitigate the extent to which bystanders dehumanised the moral transgressor. We compared three mechanisms of moral disengagement [3]. As expected, when the transgressor employed the distortion of consequences mechanism, they were dehumanised to a greater dehumanisation than when the other two mechanisms were used. We hypothesised that the moral justification mechanism would generate the lowest level of dehumanisation, yet our hypothesis was only partially supported. While moral justification did cause the least amount of dehumanisation, the displacement of responsibility mechanism had a similar effect. These findings align with prior research suggesting that both moral justification and displacement of responsibility contribute similarly to repairing trust between the transgressor and bystanders, while distorting the consequences promotes trust repair to a lesser extent [68]. Therefore, we have extended previous research by showing that moral disengagement mechanisms not only influence the degree of humanness attributed to moral transgressors but that they do so in a non-uniform way.

We also expected an interaction between incivility and moral disengagement mechanisms. Specifically, we anticipated that the use of moral justification would neutralise the effect of incivility, leading to similar levels of dehumanisation for behaviours that exhibited high and low incivility. This hypothesis was partially confirmed, as dehumanisation levels were similar for behaviours with high and low incivility on the blatant measure, but they differed on the subtle one. Specifically, only moral justification mitigated the dehumanising effect of performing highly uncivil behaviours compared to less uncivil ones. This finding was only observed on the blatant measure, however; neither mechanism reduced it on the subtle measure. These findings suggest that, regardless of the mechanism, uncivil behaviours lead to greater dehumanisation of the transgressor, highlighting the pervasive impact of incivility, at least on the subtle measure.

Lastly, we hypothesised that moral disengagement mechanisms would not only modify the effect of dehumanisation but also influence the level to which observers perceived a behaviour as severe. Confirming our hypothesis, displacement of responsibility appears to be the most effective mechanism for reducing the perceived severity of a behaviour. This mechanism redefines the behaviour by shifting responsibility away from the actor, emphasising their distance from any or all detrimental consequences of the behaviour. Therefore, congruent with literature on intentionality, if the transgressor asserts that they no intent, their action is judged as less wrong [18,69].

## General discussion

Our research makes several contributions to the literature on how bystanders perceive moral transgressors. First, the results from the two studies presented in this paper highlight the importance of incivility as a factor in the analysis of immoral behaviour. Similar to moral foundations [29,32], incivility is a cross-sectional dimension that makes it possible to study some implicit characteristics of immoral behaviour. Indeed, each immoral behaviour seems to exhibit a greater or lesser degree of incivility, just as it may violate several of the moral foundations to a greater or lesser extent. Thus, congruent with our definition of incivility, each immoral behaviour inherently embodies ‘a lack of courtesy and good citizenship’ to some extent, and this factor also impacts attributions of humanness. This finding aligns with previous research suggesting a link between incivility and lack of humanness [12,65].

It is important to highlight that in our second study, aside from the effects of the different moral disengagement mechanisms, the degree of incivility associated to the actions meaningfully impacted dehumanisation of the transgressor. The reason civility plays such an important role may be that a significant part of everyday social life in modern societies takes place in public settings inhabited by strangers [70]. In this situations, civility is the social lubricant that facilitates daily interactions [71]. We argue that the robust association between civility and humanness attributions stems from the fact that incivility indicates a disregard for others’ right to a pleasant experience in shared spaces. In this context, people infer that uncivil individuals lack self-control and concern for others [72]. Thus, the transgressor’s failure to consider others’ perspectives may help explain why transgressing civil norms leads to dehumanisation and may even explain why civility is defined as a uniquely human characteristic [46].

In addition to analysing attributions of humanness to moral transgressors based on the incivility of their behaviour, this study focused on investigating whether different moral disengagement mechanisms could impact these attributions. We hypothesised that moral justification would be the most effective mechanism in preventing bystanders from dehumanising the transgressor. Although our results show that moral justification is more effective than distorting the consequences of the action, it was similarly efficacious in reducing dehumanisation as employing the displacement of responsibility mechanism. This result was also observed when comparing different levels of incivility, as both moral justification and displacement of responsibility were more effective than distorting the consequences. Therefore, our results suggest that, compared to the other moral disengagement mechanisms, distortion of consequences is less effective for achieving a more benevolent judgement from bystanders.

We suggest that the distortion of consequences mechanism fails because it attempts to portray the behaviour as not truly wrong, even though bystanders would have already judged it as immoral. In contrast, the other mechanisms we analysed do not attempt to influence this initial moral appraisal. Instead, they focus on other aspects that may mitigate the harshness of third-party evaluations. Moral justification focuses on legitimising the severity of the action under a consequentialist approach, arguing that the negative action was done to promote a greater, morally correct goal [3,16]. Meanwhile, displacement of responsibility attempts to reduce the intentionality of the transgressor without minimising the importance of the behaviour. This mechanism would be effective because the intention of the transgressor seems key to immorality judgements, beyond the severity of the action itself [18].

### Theoretical and practical implications

Overall, our research contributes to the research on moral foundations by showing that only care and purity foundations are associated with dehumanisation. Additionally, we incorporate civility as a characteristic of immoral behaviours, demonstrating that it is linked to dehumanisation (Study 1). Our results confirm that the effect of incivility persists even when individuals employ moral disengagement mechanisms (Study 2). Only moral justification successfully neutralised the dehumanising effect of incivility when we assessed dehumanisation using the blatant measure, while neither mechanism reduced it on the subtle measure. In other words, this mechanism is only capable of altering external, overt and publicly expressed opinions, but it fails to influence internal, more prejudiced and less easily accessible judgements. Similarly, only moral justification counteracts the effect of incivility when observers assess the perceived severity of a behaviour. Despite previous research has treated the different mechanisms of moral disengagement as various means to achieve the same goal [7], our findings indicate that they are not equally effective, either in isolation or with regard to civility, indicating the need for further investigation into their relative efficacy. Additionally, these results suggest that incivility may influence dehumanisation in an intuitive way. This finding aligns with a dynamic conception of moral decision-making, which posits that the latter is carried out not only by objective reasoning but also by subjective factors, such as self-interest bias [26,73,74] or the extent to which the consequences of the immoral behaviour impact satisfactory social relationships.

These findings also have significant practical implications for various areas, including perceptions of transgressions and transgressors within justice systems, evaluation of political leaders or commercial brands. Moral disengagement mechanisms are used after moral violations and understanding their impact can provide valuable insights into how transgressors are evaluated in key contexts such as legal trials or assessments of political leaders. In this regard, our results suggest that transgressors who use moral justification or displacement of responsibility are more likely to sway public opinion in their favour, resulting in a more lenient judgement compared to those who distort the consequences of their actions. Our insights also shed light on cynical marketing strategies such as greenwashing or purplewashing, which function similarly to moral justification by enhancing public perception of brands through claims of moral action.

### Limitations and future directions

Although our research opens the door to further investigations, it is not without some limitations. First, we employed a non-discrete approach in exploring the moral foundations and incivility that underlie each behaviour in our study. Although we attempted to minimise fatigue effects by limiting the number of behaviours participants were asked to rate in Study 1, we did not use attrition measures. Consequently, we do not know the extent to which participants dropped out due to fatigue. Additionally, despite our careful selection of behaviours for Study 2, the generalisability of the results within each category likely remains limited, as only two behaviours per cell were used. For future studies, it would be beneficial to include an even larger number of behaviours to confirm the findings of our second study and broaden the spectrum of transgressions using this approach. Furthermore, it would be relevant to a context-dependent approach for stimuli. For instance, future applied research might address how immoral behaviours with different levels of incivility relate to pro-environmental conduct. Sensitisation campaigns or attitude-change initiatives could address harmful behaviours that are more closely tied to individuals’ everyday interactions and social engagement. Moreover, it could be relevant to include behaviours with a positive valence in future studies. This approach would researchers to examine whether moral behaviours with a high degree of civility lead to greater perceived humanness compared to those with a lower level of civility. Exploring these aspects would provide deeper insights into the complexities of human behaviour and morality, paving the way for more targeted and effective intervention strategies.

Another avenue for future research involves examining the impact of contextual features in how incivility is assessed. Although we did not classify our behaviours in these terms, we believe that the presence of other people can influence incivility attributions. In particular, we posit that incivility often requires a social context with other individuals present, whereas behaviours low in incivility are more likely to occur in private settings. In short, behaviours are likely to seem more uncivil to people, and we would dehumanise the transgressor more, when the behaviours directly impact their everyday social interactions. This perspective is reminiscent of the ‘not in my backyard’ (NIMBY) phenomenon [75], where individuals or groups oppose various types of development in their communities because they perceive them as dangerous or undesirable, even though they do not typically object when these facilities are installed in other neighbourhoods.

In our study, we employed a single moral disengagement mechanism for each transgressive behaviour. However, given how swiftly social interactions occur and how inherently complex they are, individuals are likely to utilise multiple explanations simultaneously. Moreover, they may even blend different types of justifications in their daily interactions. Therefore, although it would be challenging in terms of design, future research might examine how various moral disengagement mechanisms work simultaneously. Furthermore, research could study whether the effectiveness of these mechanisms is context-dependent, as some may be less effective in certain situations than in others. For instance, the mechanism of distorting of consequences may be more effective for transgressors in a conflict context. A transgressor who commits an immoral act without prior provocation and distorts its consequences may appear unscrupulous. However, in a scenario where the transgressor has been previously provoked, the action may seem more understandable, and the same mechanism could function more effectively. Such studies would promote a more complete understanding of the moral disengagement or moral rationalisation process.

## Supporting information

S1 Appendix Means (and standard deviations) of moral foundations and civility of all transgressive behaviours. (DOCX)
