# Supplementary material for: ‘It’s not just immoral!’: The role of moral disengagement and incivility in dehumanising the transgressor of immoral behaviour
Source: PLoS One. 2025 May 7;20(5):e0322212. doi: 10.1371/journal.pone.0322212 (PMC12058180; doi:10.1371/journal.pone.0322212)
Supplement: S1 Appendix — (DOCX) [file pone.0322212.s001.docx]

| **S1 Table. Means (and standard deviations) of moral foundations and civility of all transgressive behaviours** | | | | | | | |
| --- | --- | --- | --- | --- | --- | --- | --- |
| **Transgressive behaviours** | | ***Care/Harm*** | ***Fairness/Cheating*** | ***Loyalty/Betrayal*** | ***Authority/Subversion*** | ***Purity/Degradation*** | ***Civil/Uncivil*** |
|  |  | *M (SD)* | *M (SD)* | *M (SD)* | *M (SD)* | *M (SD)* | *M (SD)* |
| **Care/Harm** | Starting a false rumour about someone | 6.45 (0.96) | 4.98 (2.09) | 4.83 (1.99) | 2.83 (2.16) | 2.15 (1.95) | 5.42 (1.69) |
|  | Not helping a blind person when they ask you for help | 5.35 (1.76) | 5.03 (2.07) | 3.55 (2.40) | 3.55 (2.40) | 2.05 (1.62) | 6,75 (0.63) |
|  | Harassing someone on social media | 6.94 (0.33) | 5.97 (1.54) | 5.19 (1.86) | 5.19 (1.86) | 5.06 (2.06) | 6,33 (1.22) |
|  | Not pulling over for an ambulance that is trying to pass you | 5.84 (1.88) | 4.82 (2.14) | 4.16 (2.44) | 4.16 (2.44) | 5.71 (1.67) | 6,39 (1.36) |
|  | Spanking your child for getting bad grades in school | 6.55 (1.39) | 5.68 (1.93) | 3.79 (2.52) | 3.79 (2.52) | 2.92 (2.45) | 4,71 (2.57) |
|  | Intentionally running over an animal | 6.93 (0.47) | 5.65 (2.06) | 4.28 (2.46) | 4.28 (2.46) | 3.58 (2.64) | 5,70 (2.04) |
|  | Confronting someone on the street because you think they have looked at you | 5.32 (2.17) | 4.74 (2.31) | 2.97 (2.22) | 2.97 (2.22) | 3.44 (2.27) | 6,68 (1.15) |
|  | Loudly telling a loved one that the dinner they cooked tastes awful | 5.14 (1.58) | 3.31 (1.72) | 3.33 (2.03) | 3.33 (2.03) | 2.75 (2.01) | 5,75 (1.66) |
| **Fairness/Cheating** | Cheating at a board game | 2.31 (1.67) | 4.86 (1.90) | 3.81 (1.88) | 2.22 (1.61) | 1.39 (1.15) | 4.03 (2.12) |
|  | Stealing the tip from another table at a restaurant | 3.85 (2.13) | 5.92 (1.53) | 4.73 (2.02) | 3.68 (2.22) | 1.93 (1.79) | 6.55 (0.90) |
|  | Calling in sick when you are feeling well to avoid going to work | 2.34 (1.79) | 5.00 (1.86) | 4.82 (1.78) | 4.79 (1.93) | 1.76 (1.70) | 4.74 (2.20) |
|  | Hiring a relative for a job, instead of a more qualified applicant | 2.45 (1.86) | 5.82 (1.81) | 4.37 (2.22) | 2.29 (2.03) | 1.53 (1.25) | 3.66 (2.35) |
|  | Pretending to love someone just to have sex | 6.15 (1.25) | 4.55 (2.21) | 4.10 (2.38) | 1.68 (1.25) | 4.60 (2.22) | 4.33 (2.18) |
|  | Stealing an idea from someone at work and presenting it as yours | 5.30 (1.60) | 6.50 (0.87) | 5.88 (1.38) | 3.40 (2.16) | 1.83 (1.52) | 5.98 (1.40) |
|  | Accepting more than the correct change when making a purchase | 2.15 (1.50) | 4.50 (2.00) | 2.44 (1.71) | 2.35 (1.84) | 1.38 (1.07) | 5.79 (1.65) |
| **Loyalty/Betrayal** | Saying negative things about your place of employment | 2.71 (1.75) | 2.55 (1.81) | 3.71 (2.40) | 2.84 (2.05) | 1.68 (1.40) | 3.08 (1.96) |
|  | Badmouthing your group of friends behind their back | 5.29 (1.67) | 4.16 (2.02) | 5.84 (1.37) | 2.26 (2.02) | 2.05 (1.86) | 4.76 (1.99) |
|  | Telling family secrets when family members have asked you not to do so | 5.56 (1.66) | 4.39 (2.06) | 6.19 (1.09) | 3.89 (2.11) | 2.42 (1.92) | 4.33 (2.14) |
|  | Having sex with a friend’s significant other | 6.18 (1.45) | 4.55 (2.35) | 5.68 (1.79) | 2.70 (2.27) | 3.05 (2.34) | 4.70 (2.15) |
|  | Breaking up with your partner if they are diagnosed with a serious disease | 6.50 (0.68) | 5.15 (2.04) | 5.03 (2.38) | 1.80 (1.56) | 2.52 (2.01) | 4.48 (2.06) |
|  | Not supporting a friend when they are moving to a new place because you are going to miss them | 4.50 (2.06) | 4.05 (2.14) | 4.60 (2.01) | 1.73 (1.36) | 1.53 (1.41) | 4.80 (1.90) |
|  | Voting for a random candidate when your friend is up for election | 2.29 (1.82) | 2.35 (2.04) | 3.91 (2.12) | 1.88 (1.74) | 1.12 (0.54) | 2.76 (2.42) |
|  | Secretly dating your best friend’s crush | 5.17 (1.89) | 3.58 (1.84) | 5.50 (1.93) | 2.19 (1.62) | 1.58 (1.13) | 4.11 (1.99) |
| **Authority/Subversion** | Bringing bottles, cans, or food to an event (cinema, concerts, football, etc.) where it is prohibited | 1.61 (1.24) | 2.47 (1.68) | 1.95 (1.43) | 3.76 (1.81) | 1.61 (1.40) | 3.26 (2.02) |
|  | Arriving late for work when you know the boss won’t notice | 1.40 (0.67) | 3.52 (1.89) | 3.13 (2.10) | 3.70 (2.05) | 1.23 (0.77) | 3.07 (2.14) |
|  | Insulting the police when you see them asking someone else for their documentation | 3.84 (2.14) | 3.63 (2.29) | 3.42 (2.46) | 5.76 (1.85) | 1.84 (1701) | 5.34 (1.89) |
|  | Intentionally ignoring the teacher when they talk to you | 4.19 (2.01) | 3.92 (2.02) | 3.50 (1.98) | 5.50 (1.30) | 1.92 (1.68) | 5.42 (1.64) |
|  | Replying to the police in another language so they cannot understand you | 2.30 (1.74) | 3.75 (2.24) | 3.08 (2.02) | 6.00 (1.30) | 1.92 (1.76) | 5.65 (1.53) |
|  | Failing to stop your motor vehicle for police | 2.50 (2.00) | 3.45 (2.22) | 4.20 (2.00) | 6.75 (0.67) | 1.68 (1.54) | 5.8 (1.70) |
|  | Insulting your coach because they order you to go to the bench during a game | 4.62 (2.15) | 4.26 (2.22) | 3.62 (2.09) | 5.74 (1.66) | 1.76 (1.63) | 5.65 (2.10) |
|  | Nicknaming a teacher to laugh behind their back | 5.56 (1.56) | 3.97 (1.89) | 3.11 (2.02) | 5.53 (1.56) | 2.33 (1.88) | 5.69 (1.55) |
|  | Ignoring the curfew set by parents to get home | 2.86 (1.57) | 2.31 (1.47) | 4.14 (1.93) | 5.00 (1.62) | 1.19 (0.47) | 3.25 (1.86) |
| **Purity/Degradation** | Placing an advertisement expressing your desire to be sacrificed and devoured | 2.92 (1.86) | 2.15 (1.56) | 2.88 (2.00) | 2.07 (1.42) | 3.95 (2.22) | 5.50 (1.84) |
|  | Using someone else’s toothbrush without their permission | 2.53 (2.09) | 1.66 (1.40) | 1.63 (1.38) | 1.66 (1.42) | 5.82 (1.54) | 4.03 (2.26) |
|  | Wearing the same underwear for two weeks | 5.50 (2.06) | 4.65 (2.36) | 3.75 (2.32) | 4.92 (2.15) | 4.83 (2.66) | 6.73 (0.88) |
|  | Masturbating publicly while looking at someone | 2.61 (2.19) | 2.34 (2.07) | 3.03 (2.42) | 2.71 (2.28) | 3.84 (2.24) | 3.61 (2.59) |
|  | Making out with your sibling just for practice | 5.39 (1.91) | 3.67 (1.92) | 3.28 (2.09) | 3.72 (2.30) | 6.17 (1.34) | 4.44 (1.17) |
|  | Vomiting in the middle of a meal in order to continue eating | 4.75 (2.14) | 2.50 (1.92) | 2.63 (2.10) | 2.65 (2.29) | 6.15 (1.67) | 4.70 (2.36) |
|  | Failing to shower for a week | 1.94 (1.82) | 1.56 (1.40) | 1.56 (1.52) | 1.71 (1.71) | 3.47 (2.08) | 4.29 (2.19) |
|  | Rubbing up sexually against a stranger on public transport | 5.94 (1.98) | 5.06 (2.30) | 4.33 (2.41) | 4.25 (2.41) | 6.31 (1.69) | 6.58 (0.94) |
| **Civil/Uncivil** | Pretending you haven´t seen someone waving at you | 2.83 (1.87) | 2.70 (1.76) | 2.80 (1.90) | 2.20 (1.70) | 1.52 (1.24) | 5.43 (1.61) |
|  | Not giving way when entering or leaving an establishment | 1.97 (1.73) | 3.08 (2.17) | 2.79 (2.09) | 2.34 (2.03) | 1.71 (1.59) | 6.13 (1.61) |
|  | Not being punctual when meeting someone | 2.39 (1.37) | 4.08 (1.76) | 2.68 (1.74) | 1.87 (1.53) | 1.61 (1.33) | 5.37 (1.62) |
|  | Interrupting someone when they are speaking | 2.85 (1.73) | 3.58 (2.24) | 2.6 (1.79) | 3.28 (2.22) | 1.78 (1.59) | 5.83 (1.37) |
|  | Yawning loudly in public | 1.66 (1.60) | 1.68 (1.49) | 1.71 (1.63) | 2.82 (2.14) | 1.82 (1.57) | 4.58 (1.90) |
|  | Chewing loudly with your mouth open | 1.58 (0.84) | 1.56 (1.27) | 1.22 (0.72) | 2.08 (1.64) | 1.39 (1.13) | 5.75 (1.73) |
|  | Not paying attention to someone explaining something | 3.15 (1.93) | 3.50 (2.17) | 3.28 (1.81) | 3.57 (2.11) | 1.48 (0.96) | 5.75 (1.61) |
|  | Damaging street furniture | 4.69 (1.86) | 5.00 (1.96) | 4.19 (2.09) | 5.72 (1.54) | 2.28 (1.92) | 6.67 (0.72) |
|  | Playing loud music on public transport | 2.94 (2.07) | 3.91 (2.29) | 2.38 (1.89) | 4.15 (2.13) | 1.56 (1.19) | 6.82 (0.46) |
|  | Skipping turns in a queue | 2.07 (1.35) | 5.35 (1.87) | 2.93 (2.08) | 3.30 (2.09) | 1.33 (0.76) | 6.50 (0.91) |
|  | Not apologising when you bump into someone | 2.23 (1.37) | 2.3 (1.22) | 1.70 (1.11) | 2.20 (1.77) | 1.65 (1.07) | 6.15 (1.17) |
|  | Not saying thank you to someone who does you a favour | 3.00 (1.84) | 3.64 (2.18) | 3.00 (2.08) | 2.42 (1.92) | 1.39 (0.90) | 6.36 (1.12) |
|  | Not giving up your seat to an elderly person | 4.75 (2.07) | 5.00 (1.72) | 4.64 (2.07) | 4.03 (2.20) | 2.17 (1.76) | 6.53 (0.81) |
|  | Not picking up your dog’s droppings | 3.50 (1.92) | 4.31 (2.04) | 4.06 (2.04) | 4.89 (2.04) | 2.00 (1.51) | 6.39 (1.52) |
|  | Putting rubbish outside the bin | 3.18 (2.10) | 3.55 (2.13) | 3.34 (2.22) | 4.47 (1.81) | 1.95 (1.74) | 6.00 (1.43) |
|  | Not flushing the toilet in a public restroom | 3.05 (2.12) | 3.08 (2.14) | 2.68 (2.12) | 2.55 (2.10) | 2.92 (1.98) | 6.53 (0.89) |
|  | Throwing litter on the ground | 3.72 (2.19) | 4.13 (2.19) | 3.43 (2.36) | 4.20 (2.14) | 2.10 (2.01) | 6.82 (0.55) |
|  | Parking on the pavement | 2.58 (2.06) | 3.74 (2.32) | 3.32 (2.45) | 3.97 (2.26) | 1.53 (1.33) | 6.29 (1.01) |
|  | Not respecting the bicycle lane | 2.94 (1.89) | 3.06 (2.26) | 2.82 (2.12) | 3.88 (2.21) | 1.32 (1.04) | 6.32 (1.15) |
|  | Putting your feet up on a seat on public transport | 1.70 (1.02) | 2.43 (1.74) | 1.70 (1.20) | 3.40 (2.08) | 1.40 (0.90) | 5.67 (1.83) |
| **Neutral** | Putting a letter in the mailbox | 1.25 (0.76) | 1.31 (1.00) | 1.21 (0.72) | 1.29 (0.87) | 1.12 (0.45) | 1.62 (1.44) |
|  | Turning the pages of a book | 1.19 (0.64) | 1.22 (0.72) | 1.28 (0.83) | 1.28 (0.86) | 1.16 (0.58) | 1.43 (1.05) |

Note. The behaviours are organised according to the norm they theoretically are meant to transgress, based on previous research. Thus, the column where they theoretically belong is shaded.
